# Association Between Retinal Microvascular Metrics Using Optical Coherence Tomography Angiography and Carotid Artery Stenosis in a Chinese Cohort

**DOI:** 10.3389/fphys.2022.824646

**Published:** 2022-06-03

**Authors:** Qian Xu, Hongyi Sun, Qu Yi

**Affiliations:** ^1^ Qilu Hospital, Shandong University, Jinan, China; ^2^ Tai’an City Central Hospital, Tai’an, China

**Keywords:** retinal microvasculature, optical coherence tomography angiography, vessel density, fractal dimension, carotid intima media thickness, carotid artery stenosis

## Abstract

**Objectives:** The main aim was to investigate the association between retinal microvascular metrics using optical coherence tomography angiography (OCTA) and carotid artery stenosis (CAS) in an aging Chinese cohort.

**Methods:** In this cross-sectional and observational study, 138 eyes of 138 participants were examined. Indices of the microcirculation measured by OCTA included mean vessel density (VD), skeleton density (SD), vessel diameter index (VDI), fractal dimension (FD) and foveal avascular zone (FAZ) of the superficial retinal layer (SRL) and deep retinal layer (DRL), and peripapillary vessel caliber. The correlation of these indices with the carotid atherosclerotic lesions including carotid intima media thickness (CIMT) and common carotid artery (CCA) plaque was assessed.

**Results:** A total of 72 of 138 eyes demonstrated an increased (≥1 mm) CIMT, and 32 of the eyes presented common carotid plaques. Macular VD, SD, and FD were decreased with the increasing CCA caliber diameter (*p* < 0.05, respectively). Superficial and deep macular FDs were negatively associated with CIMT as well as the existence of CCA plaques (*p* < 0.05, respectively).

**Conclusion:** Changes in retinal microvasculature accessed by OCTA may be used as one of the non-invasive early indicators to monitor asymptomatic CAS.

## Introduction

Carotid artery stenosis (CAS) has been reported to be responsible for approximately 10–20% of all cases of ischemic strokes (Benjamin et al., 2017). It is well recognized that the prevalence of CAS increases with age, especially after age 70. Faced with the increasing epidemic incidence of asymptomatic carotid stenosis (ACS), the early diagnosis and intervention are particularly meaningful for aging populations to reduce the risk of stroke which was one of the leading causes of death and disability globally (Feigin et al., 2016). Over the past few decades, there have been significant efforts to develop inexpensive, non-invasive indicators of CAS. Carotid intima media thickness (CIMT), degree of stenosis, and plaque morphology, measured by ultrasound, have been widely regarded as a representative marker for not only stroke but also myocardial infarction, independently of traditional cardiovascular risk factors (Virani et al., 2020).

Spring from the common and internal carotid artery (ICA), and sharing many structural, vascular, and functional similarities with the brain (Cohen et al., 2010), retinal microvasculature provides a unique window into the status of brain microcirculation directly and repeatedly *in vivo*, using non-invasive optical techniques. So far, studies using fundus photography have demonstrated that retinal vessel caliber was associated with the incidence of stroke and dementia (Baker et al., 2007; McGeechan et al., 2009). Moreover, retinal vascular changes have been used as an indicator of asymptomatic carotid artery atherosclerosis (ATS) (Song et al., 2013). However, the early abnormalities of retinal microvasculature associated with CAS have been still less well established partly because previous investigations on the retinal vasculature using fundus images cannot display the retinal microvascular network in sufficient detail.

Optical coherence tomography angiography (OCTA) is a new method that provides a high-resolution imaging platform for the macular region as well as the peripapillary region of the optic nerve head with high reliability and reproducibility. With advancements of this imaging technique, microvascular information in separate layers of superficial and deep retina can be visualized quantitatively at the micrometer level (Kashani et al., 2017). A number of studies have focused on quantifying OCTA-derived vascular metrics, including flow area, vessel density (VD), skeleton density (SD), foveal avascular zone (FAZ,) and vessel diameter (Uji et al., 2017). These parameters lately have been shown to be useful markers for retinal capillary dropout. In addition to these vascular density measurements, other parameters that describe the complexity of the vasculature may also be useful in evaluating microvascular pathologic features in OCTA imaging, such as vessel diameter index (VDI) and fractal dimension (FD) (Bhardwaj et al., 2018). Now, it has become increasingly important in providing information on retinal microvasculature in several diseases such as stroke (Shi et al., 2021), Alzheimer’s type dementia (Bulut et al., 2018), and coronary heart disease (Monteiro Henriques et al., 2021). In our previous research, retinal vascular metrics obtained by OCTA were suggested to be objective and reliable parameters for predicting hypertensive microvascular damage (Xu et al., 2021). However, data on the association between the novel retinal vascular metrics measured by OCTA and carotid ultrasonographic parameters are largely lacking.

Given the more detailed imaging of each vascular layer provided with OCTA, studying the relationship between retinal microvasculature and ultrasound findings of the carotid artery may provide a new insight into the precise microvascular changes associated with CAS. In this study, we performed a quantitative analysis of macular capillary flow measured by VD, SD, VDI, FD, and FAZ, as well as peripapillary vessel diameter, to explore the correlation of OCTA-derived vascular metrics with carotid ultrasound variables.

## Methods

### Study Population

The study cohort was a subgroup of an observational study population that was carried out in Pingyin County, Jinan, Shandong, China. A total of 2,000 subjects with an age of 40+ years agreed to participate in the study and eventually completed the baseline examination. The Ethics Committees of Qilu Hospital had approved the study protocol, and all participants had given informed consent, according to the Declaration of Helsinki. Written consent was obtained from each subject.

Blood pressure and blood samples were obtained from these eligible patients. Patients with diabetes, hypertension, hypercholesterolemia, smoking habit, cerebrovascular disease, or cardiovascular disease were excluded. The participants in this study were also without use of any systemic medications. A trained ophthalmologist carried out the comprehensive ophthalmologic examination according to a standardized protocol which included the history of previous ocular diseases, trauma, or surgery, best corrected visual acuity (BCVA) recorded in the logarithm of the minimum angle of resolution (log MAR), refraction, slit lamp examination, intraocular pressure (IOP) measurement, and fundus photography. They had to have a best corrected visual acuity of 0.3 or better, IOP <21 mm Hg, cup/disk ratio <0.4, and absence of asymmetry. Additional exclusion criteria included refractive error exceeding ±6.0 diopters, presence of macular edema, previous intraocular surgery or laser photocoagulation, treatment with anti-vascular endothelial growth factor (VEGF) agents, or the sign of any other chorioretinal disease in the studied eye. Out of these subjects, a total of 138 participants who underwent both OCTA measurements and carotid artery ultrasound were recruited and evaluated for this study.

### Carotid Ultrasonography Measurements

Carotid ultrasonography examination was performed to detect the intimal medial thickness (IMT), peak systolic velocity (PSV), and end diastolic velocity (EDV) of the common carotid artery (CCA) using a 7-MHz linear transducer (Siemens ACUSON P500) in a head straight, flat supine position.

First, the structure of the vascular wall of the full length of CCA, the bifurcation, and the initial segment of ICA were observed from the cross section; atherosclerotic plaques and intravascular echo were also detected in this way. Then, the IMT of the distal segment of CCA (1–1.5 cm below the bifurcation level) and distal segment of CCA were measured by the longitudinal section. The diameter was defined as the vertical distance between the upper edge of the posterior wall of the vessel and the inferior border of the anterior wall. When vessel stenosis occurred, the residual diameter and original diameter should be measured. The thickness of IMT was defined as the vertical distance between the upper edge of the posterior wall of the vessel and the upper edge of the outer membrane. Location, morphology, integrity of the fibrous cap, and the acoustic characteristics of the plaque were observed, and the size of the plaque was measured. Intima thickening was defined as intimal-middle film thickness ≥1.0 mm; plaque was defined as focal intimal-middle film thickness ≥1.5 mm (Grant et al., 2003).

### Optical Coherence Tomography Angiography

Optical coherence tomography angiography images were obtained using the AngioVue OCTA system on the commercially available Avanti SD-OCT device (RTVue-XR; Optovue, Inc., Freemont, CA). Each B-scan was composed of 304 A-scans. Two consecutive B-scans were obtained at each raster location for a total of 608 B-scans per volumetric raster scan, which amounted to an imaging time of 3–4 s per raster scan. Two raster scans (X-Fast and Y-Fast) were obtained. A signal strength index of ≥40 was considered acceptable with respect to the quality of the images. The OCTA images were extracted from the OCT instrument and imported to publicly available ImageJ software (National Institutes of Health, Bethesda, Maryland, United States).

### Vascular Density and Fractal Analysis

**FIGURE 1:**
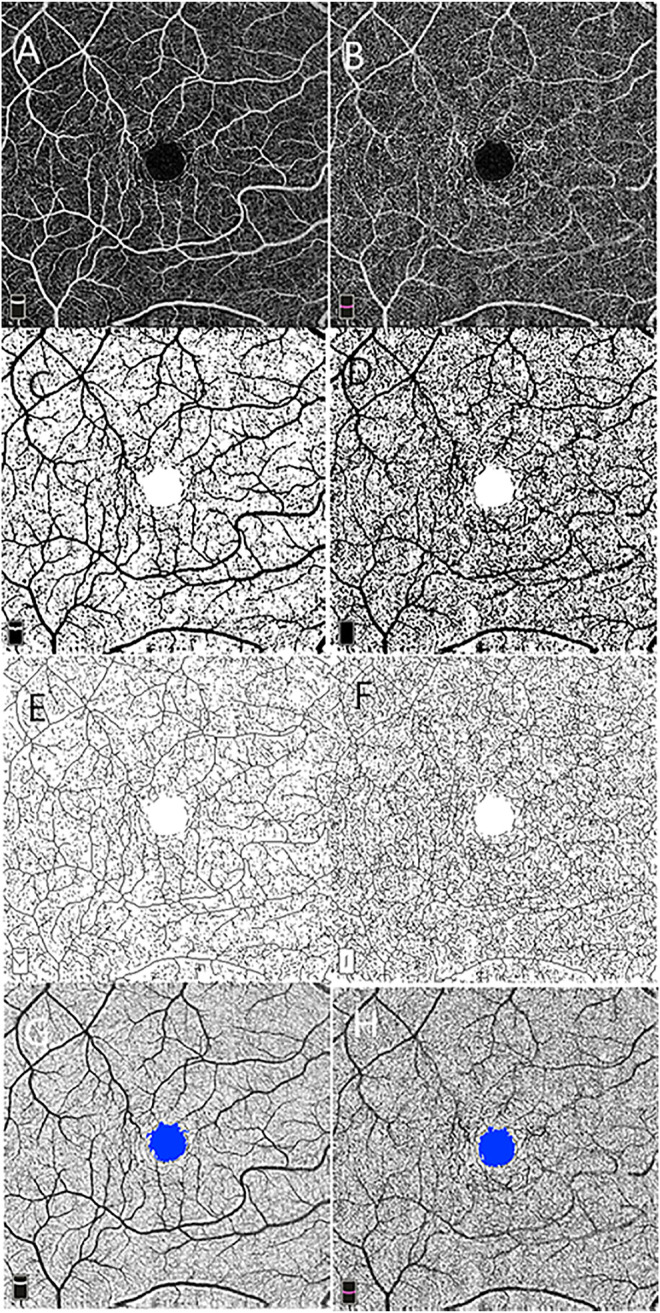
Macular vascular density measurement and foveal avascular zone (FAZ) analysis using 6 × 6 mm optical coherence tomography angiography scan centered on the macula at the level of the superficial **(A)** and deep **(B)** retinal vascular networks. Vascular density is measured in the entire contrast-enhanced binarized images of the superficial **(C)** and deep **(D)** retinal vascular layer. Skeleton density is calculated in the skeletonized images of the superficial **(E)** and deep **(F)** networks. The FAZ is shown on the superficial **(G)** and deep **(H)** networks.

Macular scans (6 × 6 mm) centered at the fovea were obtained for each subject. Optical coherence tomography angiography images of superficial and deep capillary networks were generated separately using the automated software algorithm (Figures 1A,B). Based on these default settings, the superficial retinal layer (SRL) extended from 3 μm below the internal limiting membrane (ILM) to 15 μm below the inner plexiform layer (IPL). The deep retinal layer (DRL) extended from 15 to 70 μm below the IPL. The segmentations of all examinations were checked before any measurement was performed.

Image processing and fractal analysis were conducted as described in a previous study. In brief, grayscale OCTA images of the superficial and deep capillary plexus were standardized, cropped, and binarized using a custom ImageJ macro (Zahid et al., 2016). One form represents binarization of the original scan obtained, allowing for measurement of VD (Figures 1C,D), which were calculated on the binarized images as a ratio of the area occupied by the vessel in the entire en face scan for superficial and deep networks subtracting the FAZ area. The second binary form is obtained by skeletonizing the acquired scan into 1-pixel-wide vessels allowing for measurement of SD (Figures 1E,F). SD is calculated by measuring the total vessel length in the obtained image. The VDI was calculated by using the binary blood vessel image and the skeleton image to yield the average vessel caliber in the OCTA image (pixels).

Fractal dimension (FD) as a measure of branching complexity was analyzed using the box-counting method with Fractalyse (ThéMA, Besancon Cedex, France). The box-counting method consists of dividing an image into square boxes of equal sizes and counting the number of boxes containing a vessel segment. Higher FD values reflect denser vascular branching patterns.

### Foveal Avascular Zone Measurement

Using the acquired images, the FAZ area was defined as the area inside the central border of the capillary network at the level of the superficial and deep vascular networks (Figures 1G,H).

### Retinal Vessel Caliber Measurement

**FIGURE 2:**
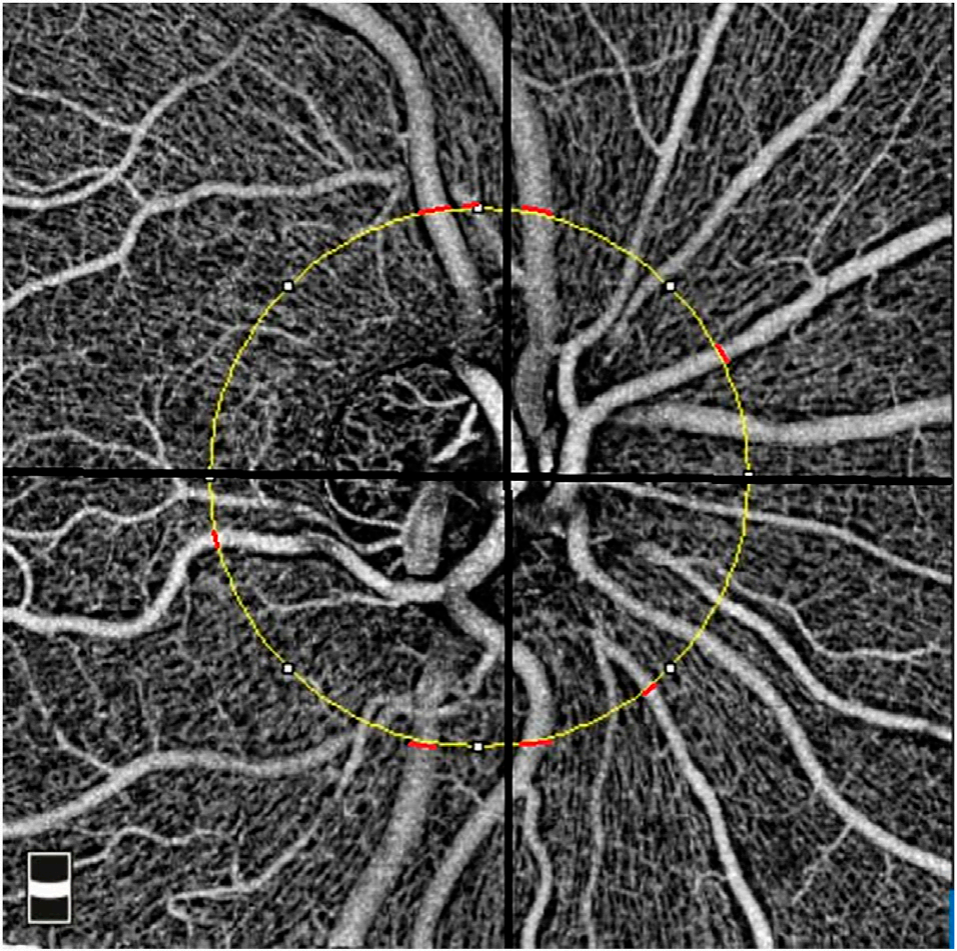
Measurement of the peripapillary vessel caliber in the 4.5 × 4.5 mm optical coherence tomography angiography image. The yellow circle represents a 3.4-mm circle centered on the disc. The location of the measurements was shown using red lines for peripapillary quadrants.

The optic nerve head (ONH) cube scan at 4.5 mm × 4.5 mm field of view was acquired. We analyzed the ONH cube scan at the retinal nerve fiber layer segmented automatically by OCTA software to measure the peripapillary vessel diameter. A circle with a diameter of 3.4 mm was centered on the ONH. For each peripapillary quadrant, the largest diameter vein and artery were marked. Two independent, trained ophthalmologists measured the width of the marked vessels at the border of the previously fitted 3.4-mm circle (Figure 2). For this purpose, an image magnification of 200× was selected. The graders selected the most appropriate image brightness and contrast to detect the borders of the vessels. The border of the vessels was manually determined using an edge-based approach (Bhargava et al., 2012).

### Statistical Analysis

Statistical analysis was performed using a commercially available statistical software program (SPSS for Mac, version 22, IBM/SPSS, Chicago, IL, USA). The data were expressed as the mean ± standard deviation (Std), and frequencies were expressed as percentages. For all statistical testing, *p* > 0.05 was considered statistically significant. To compare the groups with normal and high CIMT and groups with the absence or presence of CCA plaques, independent *t*-tests were applied. For the analysis of correlation, the partial correlation was used after adjustment for age and blood pressure. Finally, linear regression analysis was applied to investigate the association of VD, SD, VDI, FD, and FAZ of the macula, the vessel calibers around the ONH, and carotid ultrasound measurements after controlling for age, blood pressure, and gender.

## Results

Baseline characteristics and carotid ultrasound measurements stratified by sex are given in Table 1. The study included 138 eyes of 138 participants. The mean age was 59.39 ± 8.66 years (range: 40–80 years). The mean CIMT of the participants was 0.99 ± 0.27 mm. PSV (60.19 ± 17.72 cm/s vs. 58.58 ± 11.66 cm/s) and EDV (20.18 ± 6.93 cm/s vs. 19.89 ± 4.58 cm/s) were different for men and women (*p* = 0.04 and *p* = 0.02). Approximately 23.19% of the eyes presented common carotid plaques.

**TABLE 1:** Clinical characteristics and carotid ultrasonographical variables of participants, stratified by sex [mean (±Std) or n (%)].

Characteristic	All patient	Male	Female	*p*-value^a^
Age (years)	59.39 ± 8.66	60.45 ± 7.80	58.48 ± 9.30	0.08
Sex	138	64 (46.4%)	74 (53.6%)	
Common carotid artery caliber (mm)	8.06 ± 0.90	8.24 ± 0.95	7.89 ± 0.84	0.18
Common carotid intimal medial thickness (mm)	0.99 ± 0.27	1.02 ± 0.32	0.97 ± 0.21	0.68
Peak systolic velocity (cm/s)	59.33 ± 14.75	60.19 ± 17.72	58.58 ± 11.66	0.04^b^
End diastolic velocity (cm/s)	20.03 ± 5.78	20.18 ± 6.93	19.89 ± 4.58	0.02^b^
Common carotid plaques	32 (23.19%)	14 (10.14%)	18 (13.04%)	0.50

a Comparison of men and women.

b Statistical difference, *p*< 0.05.

Of the 138 eyes, thicken CIMT (≥1.00 mm) was observed in 72 eyes (52.17%). As shown in Table 2, there was no significant difference in the values of the caliber, PSV, and EDV of CCA between the two groups with normal and high CIMT (all *p* ≥ 0.05, respectively). The mean peripapillary retinal total vessel and arteriolar and venous calibers did not demonstrate a significant difference between increased and normal CIMT groups either (all *p* ≥ 0.05, respectively). Corresponding measurements in the superficial and deep macular vascular networks in eyes of all participants are also summarized in Table 2. In the vascular network of the SRL, mean VD showed margin reduction in comparison to control subjects (0.45 ± 0.06 vs. 0.42 ± 0.09, *p* = 0.03). Similarly, in both the SRL and DRL, FD of capillary plexus reduced with the increasing CIMT group when compared to eyes with normal CIMT (1.69 ± 0.02 vs. 1.68 ± 0.15, *p* = 0.01 for SRL and 1.72 ± 0.04 vs. 1.68 ± 0.04, *p* = 0.03 for DRL).

**TABLE 2:** Independent *t*-test of carotid ultrasonographical variables and optical coherence tomography angiography data between eyes with normal and thickened carotid intima media thickness.

Variable			Eyes with normal CIMT (*n* = 66)	Eyes with high CIMT (*n* = 72)	*p*-value
Carotid ultrasound measurements	CCA	Caliber (mm)	7.88 ± 0.82	8.21 ± 0.96	0.10
		PSV (cm/s)	59.11 ± 13.52	59.52 ± 15.89	0.78
		EDV (cm/s)	19.82 ± 5.79	20.21 ± 5.79	0.99
Optical coherence tomography angiography measurements	Retinal vessel calibers	Total	1.09 ± 0.14	1.10 ± 0.18	0.06
		Arteries	0.43 ± 0.79	0.44 ± 0.09	0.19
		Veins	0.66 ± 0.09	0.66 ± 0.11	0.11
	Superficial retinal layer	VD	0.45 ± 0.06	0.42 ± 0.09	0.03^a^
		SD	0.21 ± 0.31	0.20 ± 0.04	0.05
		VDI	2.11 ± 0.09	2.09 ± 0.10	0.66
		FD	1.69 ± 0.02	1.68 ± 0.15	0.01^a^
		FAZ	0.37 ± 0.14	0.39 ± 0.14	0.96
	Deep retinal layer	VD	0.49 ± 0.11	0.42 ± 0.12	0.68
		SD	0.24 ± 0.05	0.20 ± 0.05	0.75
		VDI	2.08 ± 0.09	2.04 ± 0.10	0.52
		FD	1.72 ± 0.04	1.68 ± 0.04	0.03^a^
		FAZ	0.43 ± 0.20	0.47 ± 0.17	0.92

CIMT, carotid intima media thickness; CCA, common carotid artery; VD, vessel density; SD, skeleton density; VDI, vessel diameter index; FD, fractal dimension; FAZ, foveal avascular zone.

a Statistical difference, *p* < 0.05.

The associations of CCA caliber, CIMT, and OCTA variables in different genders were evaluated after controlling for age and blood pressure, confounding variables that may be linked to CAS (Table 3). The analysis of the microvascular network geometry of different genders showed that a higher parafoveal VD and FD in the superficial capillary plexus were associated with a higher CIMT value in men (r = −0.26, r = −0.28, *p* < 0.05, respectively). When CIMT increased in women, however, OCTA variables (including VD, SD, VDI, and FD) of DRL showed a substantial decrease (r = −0.26, r = −0.30, r = −0.24, r = −0.36; *p* < 0.05, respectively), while the FAZ area did not change (*p* > 0.05). Linear regression analysis showed that the CIMT, on the other hand, only revealed an association with the mean parafoveal VD and SD of the deep retinal capillaries (β = −0.18, *p* = 0.03; β = -0.20, *p* = 0.01) but not the superficial ones ((*p*>0.05) after adjusting for age, gender, and blood pressure (Table 4). As the CIMT thickens, the FD in both SRL and DRL decreases significantly (β = −0.18, *p* = 0.03 for the SRL; β = −0.25, *p* < 0.01 for the DRL) (Table 4).

**TABLE 3:** Correlation between CCA caliber diameter, CIMT, and optical coherence tomography angiography-derived retinal vascular metrics adjusted for age and blood pressure.

Optical coherence tomography angiography variable		CCA caliber	*p*-value	CCA caliber	*p*-value	CIMT	*p*-value	CIMT	*p*-value
		Male	Female			Male	Female		
Retinal vessel calibers	Total	−0.10	0.44	0.01	0.96	−0.01	0.91	0.15	0.21
	Arteries	−0.19	0.15	0.23	0.06	−0.09	0.47	0.28	0.02^a^
	Veins	−0.16	0.90	−0.10	0.43	0.14	0.28	−0.00	0.99
Superficial retinal layer	VD	−0.22	0.09	−0.42	<0.01^a^	−0.26	0.04^a^	0.02	0.84
	SD	−0.25	0.05	−0.38	<0.01^a^	−0.24	0.06	−0.05	0.67
	VDI	0.09	0.48	−0.09	0.48	0.10	0.44	0.11	0.34
	FD	−0.09	0.49	−0.35	<0.01^a^	−0.28	0.03^a^	−0.15	0.20
	FAZ	−0.14	0.29	0.01	0.96	0.01	0.96	0.08	0.50
Deep retinal layer	VD	−0.16	0.23	−0.40	<0.01^a^	−0.18	0.17	−0.26	0.03^a^
	SD	−0.19	0.15	−0.39	<0.01^a^	−0.19	0.15	−0.30	0.01^a^
	VDI	−0.12	0.36	−0.39	<0.01^a^	−0.09	0.47	−0.24	0.04^a^
	FD	−0.14	0.28	−0.32	0.01^a^	−0.25	0.05	−0.36	<0.01^a^
	FAZ	−0.23	0.08	0.02	0.90	0.21	0.10	0.12	0.31

CCA, common carotid artery; CIMT, carotid intima media thickness; VD, vessel density; SD, skeleton density; VDI, vessel diameter index; FD, fractal dimension; FAZ, foveal avascular zone.

a Statistical difference, *p* < 0.05.

**TABLE 4:** Linear regression of ultrasonographical variables on optical coherence tomography angiography-derived data, adjusted for age, sex, and blood pressure.

Optical coherence tomography angiography variable		CCA caliber		CIMT		CCA PSV		CCA EDV	
		β	*p*-value	β	*p*-value	β	*p*-value	β	*p*-value
Retinal vessel calibers	Total	−0.03	0.71	0.54	0.50	−0.05	0.55	−0.06	0.49
	Arteries	0.03	0.71	0.07	0.38	−0.09	0.30	−0.10	0.25
	Veins	−0.04	0.60	0.06	0.43	−0.02	0.79	−0.05	0.55
Superficial retinal layer	VD	−0.26	<0.01^a^	−0.09	0.24	−0.02	0.81	−0.02	0.86
	SD	−0.27	<0.01^a^	−0.12	0.15	−0.02	0.84	0.01	0.89
	VDI	−0.01	0.93	0.10	0.19	−0.00	0.98	−0.03	0.73
	FD	−0.18	0.02^a^	−0.18	0.03^a^	−0.05	0.62	−0.06	0.49
	FAZ	−0.05	0.50	0.02	0.77	0.13	0.15	0.20	0.02^a^
Deep retinal layer	VD	−0.24	<0.01^a^	−0.18	0.03^a^	0.09	0.30	0.14	0.11
	SD	−0.25	<0.01^a^	−0.20	0.01^a^	0.09	0.34	0.15	0.09
	VDI	−0.19	0.01^a^	−0.13	0.12	0.16	0.07	0.13	0.15
	FD	−0.20	0.01^a^	−0.25	<0.01^a^	0.11	0.22	0.14	0.13
	FAZ	−0.07	0.40	0.11	0.17	0.13	0.14	0.18	0.05

CCA, common carotid artery; CIMT, carotid intima media thickness; PSV, peak systolic velocity; EDV, end diastolic velocity; VD, vessel density; SD, skeleton density; VDI, vessel diameter index; FD, fractal dimension; FAZ, foveal avascular zone.

a Statistical difference, *p* < 0.05.

For males, no correlations were observed between OCTA-derived retinal vascular metrics and CCA caliber (*p* ≥ 0.05, respectively). Unlike males, females with a narrower CCA caliber had a greater parafoveal VD and SD in both of the capillary plexus layers, which was statistically significant (r = −0.42, r = −0.38, for SRL; r = −0.40, r = −0.39, for DRL; *p* ≤ 0.01, respectively). Women’s FD values were found to be inversely related to CCA caliber (r = −0.35 in SRL, r = −0.35 in DRL, *p* ≤ 0.01, respectively). The linear regression analysis demonstrated a significant negative correlation between VD and SD of the two retinal capillary plexuses in the macular area and CCA caliber when age, gender, and blood pressure were adjusted (β = −0.26, β = −0.27, for SRL; β = −0.24, β = −0.25 for DRL; *p* < 0.05, respectively) (Table 4). The FD in both SRL and DRL decreases significantly when the CCA caliber narrows (β = −0.18, *p* = 0.02 for the SRL; β = −0.20, *p* = 0.01 for the DRL) (Table 4).

Superficial macular VD and SD was found statistically significantly lower in the eyes of subjects with the presence of CCA plaques, with respect to controls (0.38 ± 0.01 vs. 0.44 ± 0.07 for the VD; 0.18 ± 0.01 vs. 0.21 ± 0.01 for the SD; *p* < 0.01), so was the VD and SD in the DRL (0.40 ± 0.15 vs. 0.46 ± 0.12 for the VD; 0.19 ± 0.01 vs. 0.23 ± 0.01 for the SD; *p* < 0.01) (Table 5). Table 5 further indicates that when CCA plaques appeared in the eyes, macular FD at both the SRL and DRL decreased (1.67 ± 0.01 vs. 1.71 ± 0.01 for the SRL; 1.67 ± 0.01 vs. 1.71 ± 0.01 for the DRL; *p* < 0.01).

**TABLE 5:** Quantitative analysis and comparisons with an independent *t*-test of optical coherence tomography angiography-derived data between groups with the absence or presence of carotid plaques.

Optical coherence tomography angiography variable		Eyes without carotid plaques (*n* = 106)	Eyes with carotid plaques (*n* = 32)	Independent *t*-test
				*p*-value
Retinal Vessel calibers	Total	1.08 ± 0.02	1.14 ± 0.02	0.08
	Arteries	0.44 ± 0.09	0.45 ± 0.07	0.46
	Veins	0.65 ± 0.11	0.68 ± 0.08	0.17
Superficial retinal layer	VD	0.44 ± 0.07	0.38 ± 0.01	<0.01^a^
	SD	0.21 ± 0.01	0.18 ± 0.01	<0.01^a^
	VDI	2.11 ± 0.01	2.08 ± 0.04	0.38
	FD	1.71 ± 0.01	1.67 ± 0.01	<0.01^a^
	FAZ	0.37 ± 0.01	0.41 ± 0.02	0.18
Deep retinal layer	VD	0.46 ± 0.12	0.40 ± 0.15	<0.01^a^
	SD	0.23 ± 0.01	0.19 ± 0.01	<0.01^a^
	VDI	2.06 ± 0.01	2.05 ± 0.02	0.51
	FD	1.71 ± 0.01	1.67 ± 0.01	<0.01^a^
	FAZ	0.44 ± 0.02	0.49 ± 0.03	0.14

VD, vessel density; SD, skeleton density; VDI, vessel diameter index; FD, fractal dimension; FAZ, foveal avascular zone.

a Statistical difference, *p* < 0.05.

## Discussion

In this study, we quantitatively evaluated the retinal microvascular parameters using a high-resolution retinal imaging modality known as OCTA and demonstrated the negative association of macular VD, SD, FD, and deep retinal VDI in eyes and CCA caliber diameter in women but not men. The gender difference in retinal microvascular perfusion changes resulting from decreased carotid arterial blood supply was in line with previous studies of coronary and cerebral microvascular diseases (Pries et al., 2015; Faber et al., 2017). It has been hypothesized that sex-specific differences in cardiovascular disease were related to sex differences in microvascular architecture and metabolic reaction (Huxley and Kemp, 2018). Our study showed that modulation of microcirculation played more important roles in women than men to regulate tissue blood perfusion.

There are also sex-related differences in the correlation of these retinal microvascular metrics and CIMT: in males, CIMT is inversely correlated with VD and FD of SRL while in females, with VD, SD, VDI, FD and decreasing retinal arteriole diameter. According to previous studies, retinal arteriolar caliber narrowing may serve as a marker of coronary microvascular disease (Wang et al., 2008). However, it also has been represented that retinal arteriolar narrowing was related to the risk of coronary heart disease in women, not men (Wong et al., 2002). Interestingly, our study elucidated the layer-related variations associated with cardiovascular risk. Using OCTA, we could give more insights into microvascular abnormalities in different layers caused by clinical cardiovascular events. Likewise, for men, superficial macular signs were related to CAS but for women were microvascular metrics in the deep layer. The discrepancy is likely due to differences between males and females in the cardiovascular risk factors affecting CIMT (Uchmanowicz et al., 2016).

After exclusion of other possible confounding factors, macular VD and SD of the superficial and deep capillary plexus in eyes increased with CCA caliber narrowing. However, in the same participants, we did not find any significant relationship between the value of CCA caliber diameter and superficial VDI on the macula. Vessel area and length density represent the relative value of the occupied area and total length of vessels, respectively (Seaman et al., 2011). It is clearly evidenced that because of the high metabolic demand, the regulatory ability of retinal circulation is vital and essential for the macular region (Yu et al., 2005). In the subjects with narrower CCA, the capillary length of the macular capillary network seems likely to be higher to maintain stable adequate capillary flow instead of capillary vessel size, which is a pivotal finding in contrast with previous reports. This may be seen as the first step in the analysis of the macular microvascular network changes in response to alterations in CCA caliber diameter.

The ultrasound-based measurement of CIMT representing the true biological thickness of the vessel wall has been used as a marker for assessing the progression of CAS (Smith et al., 2000). Thickened CIMT combined with the existence of relevant carotid plaques, measured in the CCA, has been widely used as a powerful and independent indicator of clinical cerebrovascular and cardiovascular events (Willeit et al., 2020). The present study showed that the presence of CCA plaques was negatively associated with VD and SD of the whole retina, while CIMT was negatively associated with deep retinal VD and SD, which have not been previously demonstrated. Accumulating evidence suggests that CIMT and CCA plaques represent distinct phenotypes or different phases of atherosclerosis (Rundek et al., 2015). Our study indicated that the quantification of the retinal microvasculature in the deep retinal capillary plexus had higher discriminating powers than that in the superficial capillary layer to detect the microvascular dysfunction caused by systemic risk factors that contribute to CAS. Consistently, a previous study reported that the maintenance of sufficient VD of DCP was a protective factor of stroke (Shi et al., 2021). In addition, vessel density of the deep but not superficial retinal plexus was decreased in patients with silent cerebral infarctions (Thangamathesvaran et al., 2022). The superficial plexus is composed of not only capillaries but also retinal arterioles and venules, while deep macular vascular plexus where the plexiform layers are located is a uniform capillary network of the largest oxygen consumption. There is evidence that retinal capillary organization is an independent neurovascular unit at each capillary plexus where alteration can occur independently. In agreement, it has been reported that the retinal autoregulatory response to hyperoxia affects only the deep capillary plexus (Hagag et al., 2018). The deep vascular plexus of the retina is mainly a low-pressure network receiving blood from branches of the central retinal artery, so lumen obstruction of the carotid artery and perfusion pressure reduction could primarily affect DCP. Further investigations are needed on the mechanism of layer-related variations of the macular vascular density decrease. In addition, given the more detailed imaging of each vascular layer in the macula, our study demonstrated that CAS impairs the macular vascular architecture by affecting the skeleton of the vessel in the microvascular bed instead of capillary vessel size.

Importantly, we found macular FD in the superficial and deep retinal capillary layer was negatively associated with CIMT as well as the presence of CCA plaques. FD is a non-integer value that describes the intrinsic shape of an object which has shown its potential in characterizing the growth of tissues (Lennon et al., 2015). FD of the vascular tree is a measure of scale invariant vascular-branching pattern that has been applied for evaluating the microvascular perfusion of muscle (Kalliokoski et al., 2003), myocardial ischemia (Michallek et al., 2022), skin (Byers et al., 2018), mesentery (Wang et al., 2019), and tumor angiogenesis (Di Ieva et al., 2007; Ternifi et al., 2021). FD of retinal microcirculation is a global measure of the natural self-similar patterns of retinal vascular architecture which would disrupt by microvascular dropout or a loss of smaller caliber vessels. In many clinical studies, the retinal vascular FD is a measure of the geometric complexity of the vascular tree that quantitatively summarizes the amount of space or density that a particular object occupies (Liew et al., 2011; Huang et al., 2016). Reduced vascular FD of the retina indicates vessel rarefaction and collapse (Cheung et al., 2012). Lower retinal FD in color fundus photographs and fluorescein angiographic images has been employed as a tool in retinal angiography and demonstrated to have an inverse correlation with the progression of hypertension (Sng et al., 2013), chronic kidney disease (Sng et al., 2010), stroke (Kawasaki et al., 2011), and Alzheimer’s disease (Cheung et al., 2014). However, measuring macular FD by OCTA could describe a more detailed image of retinal microvascular architecture, especially the loss of smaller vascular branching density *in vivo*. Clinical studies have demonstrated a stronger association between FD and cardiovascular risk than the central retinal arteriolar equivalent (Zhu et al., 2014). The reduction of the OCTA-derived FD value may become a more sensitive and useful indicator of microvascular damage due to, but not limited to CAS.

To the best of our knowledge, our study is the first investigation to describe the relationship between quantitative microvascular features of the retina using OCTA and CIMT and carotid artery plaque using high-resolution ultrasonography, which can reflect the detailed CAS-related alterations in the structure of microcirculation in the retina. In addition, sharing similar anatomical features and physiological properties, the retinal vessels offer a unique site for studying the brain vascular imaging modalities *in vivo* which could not be acquired directly and repeatedly using traditional examination methods. Our findings may help to understand possible underlying mechanisms of developmental biology in ATS.

Several limitations of the current study are needed to be noted. First, this study only included a relatively small sample size. Furthermore, OCTA imaging is also prone to accompany by artifacts including automated segmentation errors and projection artifacts. It would be of interest in the future to analyze the retinal vascular changes in CAS patients by carrying out larger prospective studies.

In conclusion, the present study shows a close association between commonly used ultrasonographical parameters of carotid arteriosclerosis and microvascular changes on the macular region. Significantly lower microvascular density and reduced complexity of capillary perfusion, instead of narrowing of the retinal arterioles, may constitute as early indicators of carotid atherosclerotic lesions. Retinal vascular image screening with OCTA have great potential to become one of non-invasive early indicators to monitor asymptomatic CAS. The present data also suggest that appropriate intensive management of carotid disease may improve retinal microcirculation.

## Data Availability

The original contributions presented in the study are included in the article/Supplementary Material; further inquiries can be directed to the corresponding author.

## Abbreviations

ACS, asymptomatic carotid stenosis; ATS, artery atherosclerosis; BCVA, best corrected visual acuity; CAS, carotid artery stenosis; CCA, common carotid artery; CIMT, carotid intima media thickness; DRL, deep retinal layer; EDV, end diastolic velocity; FAZ, foveal avascular zone; FD, fractal dimension; ICA, internal carotid artery; ILM, internal limiting membrane; IMT, intimal medial thickness; IOP, intraocular pressure; IPL, inner plexiform layer; OCTA, optical coherence tomography angiography; PSV, peak systolic velocity; SD, skeleton density; SRL, superficial retinal layer; VD, vessel density; VDI, vessel diameter index.
